# Health-Promoting Properties of Selected Cyclitols for Metabolic Syndrome and Diabetes

**DOI:** 10.3390/nu11102314

**Published:** 2019-09-30

**Authors:** Tomasz Antonowski, Adam Osowski, Lesław Lahuta, Ryszard Górecki, Andrzej Rynkiewicz, Joanna Wojtkiewicz

**Affiliations:** 1 Department of Pathophysiology, School of Medicine, Collegium Medicum, University of Warmia and Mazury, 10-082 Olsztyn, Poland; adam.osowski@uwm.edu.pl; 2 Department of Plant Physiology, Genetics and Biotechnology, University of Warmia and Mazury in Olsztyn, 10-229 Olsztyn, Poland; lahuta@uwm.edu.pl (L.L.); rigorecki@gmail.com (R.G.); andrzej.rynkiewicz@uwm.edu.pl (A.R.); 3 Department of Cardiology and Cardiosurgery, School of Medicine, Collegium Medicum University of Warmia and Mazury, 10-082 Olsztyn, Poland

**Keywords:** *myo*-inositol, D*-chiro*-inositol, D-pinitol, metabolic syndrome, diabetes

## Abstract

Cyclitols play a particularly important role in cell functioning because they are involved in ion channel physiology, phosphate storage, signal transduction, cell wall formation, membrane biogenesis, osmoregulation and they have antioxidant activity. They are involved in the cell membranes as a phosphatidyl *myo-*inositol, an inositol triphosphate precursor, which acts as a transmitter that regulates the activity of several hormones, such as follicle-stimulating hormone, thyrotropin, and insulin. The aim of this paper is to characterize the selected cyclitols: *myo*-inositol, D-*chiro*-inositol, and D-pinitol in type-2 metabolic syndrome and diabetes treatment. Results and discussion: Cyclitols have certain clinical applications in the treatment of metabolic syndromes and are considered to be an option as a dietary supplement for the treatment or prevention of gestational diabetes mellitus and type-2 diabetes. Improved metabolic parameters observed after using cyclitols, like *myo*-inositol, in the treatment of polycystic ovary syndrome and type-2 diabetes suggest that they may have a protective effect on the cardiovascular system. Pinitol, together with *myo-*inositol,maybe responsible for improving lipid profiles by reducing serum triglyceride and total cholesterol. Pinitol is also well-researched and documented for insulin-like effects. *Myo-*inositol, D*-chiro*-inositol, and D-pinitol indicate a number of therapeutic and health-promoting properties.

## 1. Cyclitols

**Figure 1:**
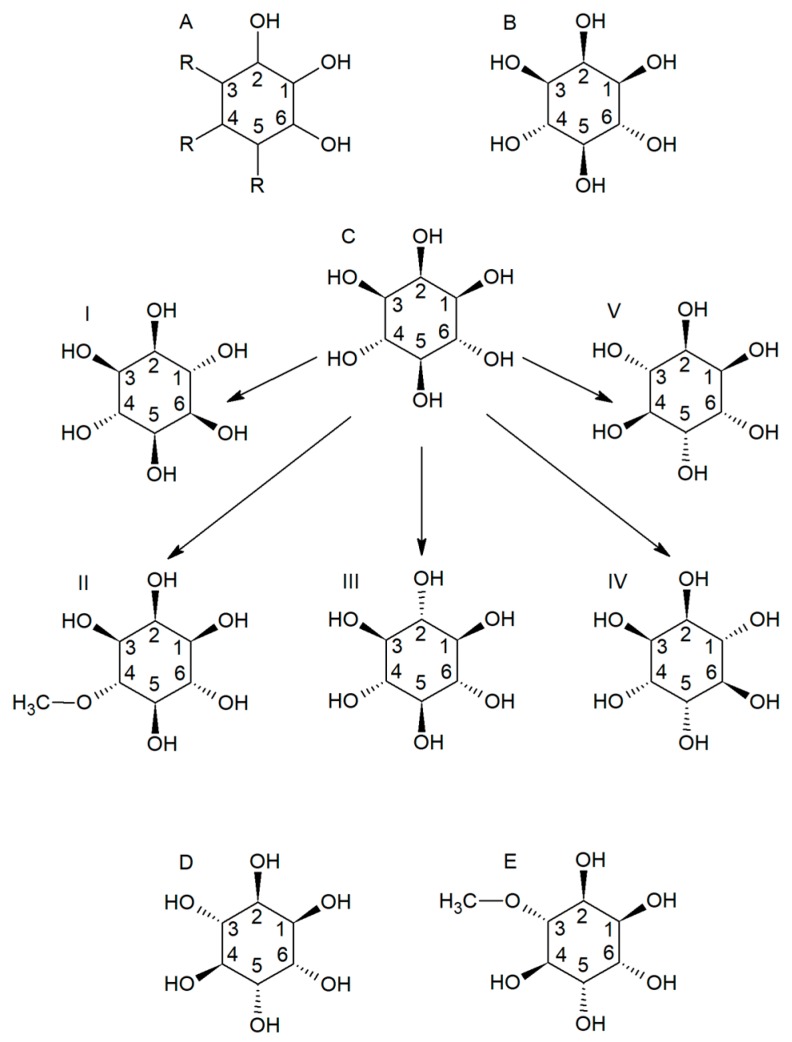
Structural formula of: (**A**) cyclito, (**B**) *Myo*-inositol, (**C**) *Myo*-inositol and its derivatives ((**I**) *Muco*-inositol, (**II**) D-ononitol, (**III**) *Scyllo*-inositol, (**IV**) *Chiro*-inositol, (**V**) *Neo*-inositol), (**D**) D-*chiro*-inositol, (**E**) D-pinitol.

The beneficial effects of many natural substances in plant products on the human body have been known for centuries. Studies from recent years have shown that increased consumption of vegetables and fruits can reduce the risk of developing many diseases [1]. Since cyclitols are cycloalkanes with one hydroxyl group on each of three or more ring atoms, they are also called polyols or sugar alcohols (Figure 1A). Cyclitols are attracting increasing attention because they are widespread in the world of plants and have a wide range of biological activities. They play a particularly important role in cell functioning because they are involved in signal transduction, membrane biogenesis, cell wall formation, ion channel physiology, phosphate storage, osmoregulation, have antioxidant activity and are one of the compatible soluble substances that are formed in the plant in response to salt or water stress [2,3].

Inositols are the most common cycloalkanes in eukaryotic cells, with the empirical formula C_6_H_12_O_6_ (1,2,3,4,5,6-cyclohexanol). These chemical compounds exist in nine possible stereoisomers. Five of them, *myo-, scyllo-, muco-, neo*- and D-*chiro*-inositol, are naturally occurring, while the other four possible isomers, L*-chiro-, allo-, epi-*and *cis-*inositol come from *myo-*inositol (MI) [4]. Inositols are involved in the cell membranes as a phosphatidyl MI (an inositol triphosphate precursor) which acts as an activity regulator of several hormones, such as follicle-stimulating hormone, thyrotropin, and insulin [5,6].

While the intracellular content of inositol is almost (> 99%) filled up by MI in most tissues [7], significant differences have been noted in the concentrations of MI and D-*chiro-*inositol (DCI), another important stereoisomer in adipose tissue, muscles, and liver. The various functions that these two isomers perform in tissues are reflected by their diversified distribution and different concentrations actively maintained in cells [8,9]. Improved metabolic parameters observed after inositol treatment in the PCOS and T2DM suggest that these compounds may have a protective effect on the cardiovascular system [10,11].

## 2. *Myo-*Inositol

MI is the most popular of all known cyclitol isomers (Figure 1B). MI is an inositol isoform and belongs to the vitamin B complex (Figure 1C). Its properties are similar to insulin and it acts as a second messenger in the intracellular insulin pathway. This molecule is involved in increasing the insulin sensitivity of various tissues to improve metabolic and ovulatory effects [12]. Additionally, MI increases cellular glucose uptake. Inositol is mainly catabolized by the kidneys, although it is excreted with urine only in small amounts [13,14]. It is also enzymatically converted to DCI by the NADH-dependent epimerase and this enzymatic reaction is stimulated by insulin. MI is necessary to ensure the proper maturation of the oocytes [8,9]. This inositol has gained popularity in clinical reproductive practice in recent years. Since the main treatment of PCOS involves an insulin sensitizer, inositol is mainly used in chronic treatment of this disease [15]. Additionally, it has been proposed as a preventive agent in neural tube defects (NTD) in patients resistant to folic acid [6,16].

Deficiencies or abnormalities in inositol metabolism interfere with glucose uptake, cause defects in areas associated with insulin resistance and long-term microvascular complications associated with diabetes [17]. This compound is present in all eukaryotic cells and acts as a structural basis for a number of transmitters. It is also an important component of structural lipids, such as phosphatidyl inositol [18]. MI induces the conversion of glucose to glycogen (stored inside the cells) and also modulates the activation of glucose transporters (GLUT) and their use. It is also important that glycogen synthesis proceeds under the control of DCI [14,19]. MI and DCI differently compensating some metabolic deregulations according to insulin resistance, e.g., phosphoinositol-3-phosphate (derived from MI) increases glucose transport in cells by stimulating GLUT4 translocation into the cell membrane. A derivative, inositol phosphor glycan (MI-IPG), also plays a key role in reducing the release of free fatty acids (FFA) from adipose tissue and inhibiting the adenylate cyclase [10]. It is also known that FFA reduces glucose removal, causing insulin resistance and increased triglyceride synthesis [20].

MI toxicity has not yet been directly investigated. However, several studies have been carried out to investigate MI effectiveness in preventing pathological changes associated with experimental diabetes (and other pathological models) and to evaluate MI’s chemopreventive potential in cancer treatment [6,16,21]

## 3. D*-Chiro-*Inositol

DCI is a form of inositol that has been found to have insulin-like properties, acting as a secondary messenger in the intracellular insulin pathway (Figure 1D). This molecule increases the insulin sensitivity of many various tissues and improves metabolic and ovulatory effects [22,23,24]. DCI plays various physiological roles. It is critical in glycogen synthesis and increases cell glucose uptake. High DCI levels are found in glycogen storage tissues, such as fat, liver, and muscles [25]. DCI also increases the level of pyruvate dehydrogenase, leading to the production of ATP in the Krebs cycle [20].

DCI is structurally related to phosphatidyl inositol phosphates, which participates in the insulin signaling pathways and stimulates glucose transport [26]. Decreased excretion of DCI in urine has been observed in rhesus monkeys and in patients with impaired glucose tolerance, insulin resistance, and T2DM [27,28].

Both MI and DCI promote glycogen synthesis, inducing the conversion of glucose-to-glycogen [29]. In mammals, MI can be converted by epimerase in *L-* or DCI. Earlier studies have shown that the volume of this inositol in urine is changed in patients with T2DM [30]. It has been also classified as insulin-sensitizing [21,31].

## 4. D*-*Pinitol

D-pinitol (called 3-O-methyl-D-chiro-inositol; DP) occurs in plants in the form of two enantiomers. DP is a DCI methyl ether, which can be found in large amounts in soybean and legume foods. This compound can make up about 1% of the dry matter of soybean meal. It is an active, low molecular weight cyclitol separated from the soybean seed coats, cotyledons and embryos (Figure 1E) [32]. It has been suggested that DP has multifunctional properties, including anti-inflammatory action, prevention of cardiovascular diseases and reducing airway allergic inflammation as well as anticancer activity [33,34,35]. Furthermore, DP has been also isolated from the leaves of *Abieswebbiana* and its anti-inflammatory effect was exhibited [36,37].

D-Pinitol can be found in peanuts, *Bougainvillea spectabilis,* and *Argyrolobiumroseum*, but for pharmaceutical purposes, it is mainly extracted from soybeans and locust beans. Its derivative, MI-IPG, plays a key role in reducing the release of FFA from adipose tissue by inhibiting the adenylate cyclase enzyme [38,39].

## 5. Metabolic Syndrome

Metabolic syndrome is characterized by a combination of the most dangerous risk factors that often lead to myocardial infarctions: Diabetes, obesity, high cholesterol, and high blood pressure. This illness is currently considered to be the main cause of the new epidemic of cardiovascular diseases [8,29,40].

Metabolic syndrome, caused by increasing obesity and a sedentary lifestyle, is a serious and increasing clinical challenge worldwide. It leads to a five-fold increase in the risk of developing T2DM and a two-fold increase of cardiovascular disease risk within 5 to 10 years. Additionally, patients with metabolic syndrome are exposed to a two- to four-fold increased risk of stroke, a three- to four-fold increased risk of myocardial infarction and a two-fold risk of death in comparison with those without the syndrome, regardless of the previous history of cardiovascular events. Metabolic syndrome is treated as a risk factor of atherosclerotic complications and its presence should be considered as a long-term risk indicator [41,42].

Metabolic syndrome was first named when Johan Kylin demonstrated a correlation between high blood pressure (hypertension) and high blood glucose (hyperglycemia) [43]. The prevalence of metabolic syndrome ranges from 10% to 84% worldwide, depending on the region, environment and studied population’s composition (gender, age, race, and ethnicity) [44]. In general, the International Diabetes Federation estimates that one-quarter of the adult population in the world may suffer from metabolic syndrome [26]. Higher socio-economic status, sedentary lifestyle and higher body mass index (BMI) were significantly related to the occurrence of this syndrome. It was also concluded that differences in human genetics, hygiene, and education affect the frequency of metabolic syndrome and its symptoms [21,45,46].

The National Health and Nutrition Examination Research Survey conducted in the United States showed a 5%, 22%, and 60% metabolic prevalence increase among patients with normal weight, overweight, and obese, respectively. The probability of metabolic syndrome occurrence increases with age (10% in patients aged 20–29, 20% in patients aged 40–49 and 45% in those aged 60–69). The metabolic syndrome ranges from 8% to 43% in men and from 7% to 56% in women worldwide [24,46]. Additionally, it has been demonstrated that there is a high prevalence of this disease among postmenopausal women, which ranges from 32.6% to 41.5%. Based on the report from the Framingham Heart Study, an increase in weight ≥2.25 kg over a 16-year period was associated with an increased risk of developing metabolic syndrome to 45% and it was demonstrated that each additional 11 cm of waist circumference enhances the risk of developing this disease within five years by 80%. Simultaneous metabolic changes are more frequent than could be expected and with the convergence of several factors, the risk of cardiovascular diseases increases 29].

## 6. Diabetes

Diabetes mellitus, one of the oldest diseases known to man, is a common metabolic disorder characterized by chronic hyperglycemia due to impaired insulin secretion and/or its action [47]. This ultimately leads to improper regulation of carbohydrates, proteins, and lipids metabolism, which contributes micro- and macrovascular complications [18,48]. The prevalence of diabetes mellitus is alarmingly rising worldwide, and the total number of patients with diabetes is expected to double from approximately 171 million in 2000 to 366 million in 2030. Countries with the highest number of patients with diabetes are India, China, and the USA. The prevalence of diabetes in India is predicted to increase from 40.9 to 69.9 million by 2025, unless necessary preventive steps are taken [49,50].

### Type-2 Diabetes

**Figure 2:**
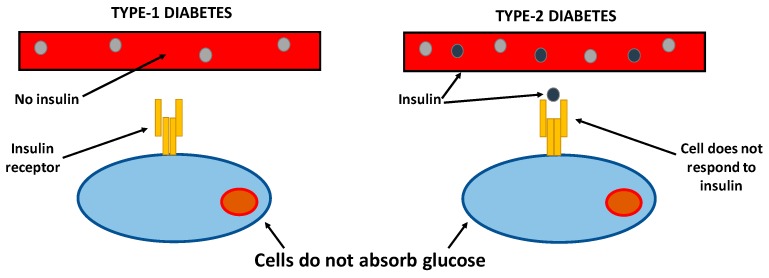
Types of diabetes.

T2DM is a disease characterized by the insulin resistance of peripheral tissues such as skeletal muscle, white adipose tissue, and the liver. To monitor diabetes, blood glucose levels must be under control, especially in the postprandial condition, because insulin regulation inhibits glucose synthesis in the liver and stimulates glucose uptake in skeletal muscles and adipose tissue [51]. T2DM was first described in 1988 as a part of the metabolic syndrome. T2DM (formerly known as diabetes without insulin) is the most common diabetes’ form, characterized by hyperglycemia, insulin resistance, and relative insulin deficiency (Figure 2) [52,53].

The number of patients with T2DM has increased in each country, with 80% of patients with this disease living in low- and middle-income countries. It is estimated that 439 million patients will have T2DM before 2030. The frequency of T2DM varies according to geographical region and risk factors related with the lifestyle and environment impact [52,54]. An increase in the number of T2DM cases is expected over the next two decades and a significant part of this increase will occur in developing countries where the majority of patients are between 45 and 64 years old [53].

**Figure 3:**
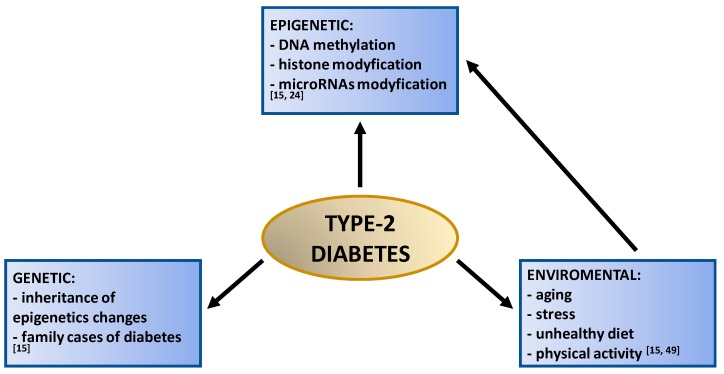
Type-2 diabetes risk factors.

T2DM is mainly caused by genetic factors (Figure 3). Many of them are also associated with human lifestyle such as: Lack of physical activity, sedentary lifestyle, smoking, and excessive alcohol consumption. It has been shown that obesity contributes to approximately 55% of T2DM cases [51,52,55]. Moreover, additional factors that increase the risk of T2DM are aging and high-fat diet [51]. There are also many diseases that can cause or exacerbate T2DM. These include obesity, hypertension, combined hyperlipidemia, and metabolic syndrome. Other causes include acromegaly, Cushing’s syndrome, thyrotoxicosis, pheochromocytoma, chronic pancreatitis, pancreatic cancer, and drugs [15].

T2DM is characterized by pancreatic beta-cell insufficiency. In young patients (from 7 to 10 years old), the loss of beta-cells is faster, which may explain the failure of treatment at an early age. This pathology leads to a reduction in the transport of glucose to the liver, muscle cells, and fat cells [52,55]. Additionally, the involvement of impaired alpha-cell functions has recently been recognized in the pathophysiology of T2DM. As a result of this dysfunction, the concentration of glucagon and glucose in the liver is not suppressed after a meal. Hyperglycemia occurs due to insufficient insulin levels and increased insulin resistance [30].

The organs involved in the development of T2DM include the pancreas (beta cells and alfa cells), liver, skeletal muscles, kidneys, brain, small intestine, and adipose tissue. Additionally, changes in the colon microbiome, dysregulation of the immune system, and inflammation have been proven to be important pathophysiologic factors and have been identified as possible therapeutic targets. Other mechanisms of the development of micro- and macrovascular complications caused by hyperglycemia are endothelial dysfunctions, advanced glycation, hypercoagulability, increased platelet reactivity, and hyper-suppression [18]. The most effective therapeutic strategies for patients with T2DM include both aspects of the complex interaction between genotype and the phenotype, although further research is needed for optimization and personalization of treatment. Patients living with T2DM are more vulnerable to various forms of complications that often lead to premature death. Generally, a tendency toward increased morbidity and mortality is observed in patients with T2DM because of its often asymptomatic onset and late diagnosis [51,53,55].

## 7. Cyclitols in Treatment

Some inositol derivatives have insulin mimicking activity and are effective in lowering blood glucose [7]. For example, the administration of DCI in rats, rhesus, mice, and humans with diabetes, increases glucose removal and improves insulin sensitivity [55]. DP is also one of the naturally occurring inositol derivatives [19,56]. It has also been reported that DP from soybean leaves and *Bougainvillaspectabilis* reduces blood glucose levels in rats with streptozotocin-induced diabetes mellitus (STZ) and in human patients with diabetes [57]. Additionally, the administration of a high dose of MI to diabetic monkeys was also effective in reducing blood glucose levels [58]. Recent studies have shown that the oral administration of MI is able to improve glucose tolerance and induce to GLUT-4 translocation in skeletal muscles in C57/BL6 mice, suggesting improvement of insulin sensitivity by inositol (Table 1) [20,59].

### 7.1. Pinitol

**Figure 4:**
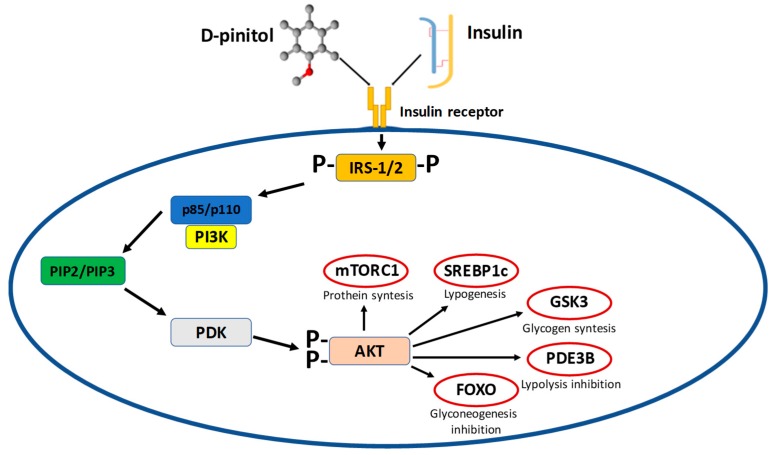
Insulin-like effect of pinitol.

It was found that a substance with a structure similar to pinitol with an undisclosed structure obtained from *Bougainvillea spectabilis* reduces the blood glucose levels in mice with normal insulin deficiency. In an animal model of diabetes, DP has been described as an anti-diabetic drug, with an insulin-like effect and the ability to enhance insulin activity by translocation of the GLUT in the mice skeletal muscles [52,63]. In patients with T2DM, treatment with pinitol improved glycemic control, decreased the level of adipocytokines, and decreased metabolic parameters associated with the risk of cardiovascular diseases [62]. In the cardiovascular system, DP was able to prevent diabetic-induced endothelial dysfunctions in the mesenteric artery. This protective effect was assigned to the antioxidant ability, which was proven to be responsible for maintaining the nitric oxide signaling. However, the mechanism by which DP induces anti-hyperglycemic properties is still unknown [64,65]. Moreover, there have been no studies confirming the anti-hyperglycemic properties of DP (Figure 4). Furthermore, natural remedies have gained much attention due to their various biological activities, such as anti-inflammatory and antioxidant properties. All of these functions are related to the ability of pinitol to attenuate or suppress oxidative stress and inflammation, both in vitro and in vivo [66,67].

In a study conducted in 2005, 30 patients with T2DM were treated with hypoglycemic agents using a recommended diet and physical exercises. In this treatment, 95% pure pinitol obtained from soy was used. Patients were randomly assigned to receive an oral dose of 600 mg of pinitol or a placebo consisting of lactose twice a day for 13 weeks. They were asked to avoid legumes and citrus fruits, but not to change their usual medicines, eating habits, or lifestyles. They were also recommended a diet which should be maintained in the guidelines: 30–35 kcal/kg ICC, 60% carbohydrate, 20% protein, and 20% fat and physical activity for 30 min of moderate exercises every other day. Correctness in the use of exercise and nutrition programs, standard medication, and the frequency of potential side effects such as hypoglycemia, abdominal pain, bloating, nausea, diarrhea, and allergic symptoms, were monitored every week by a questionnaire. Anthropometric and biochemical measurements and evaluation of food intake with the 24-h withdrawal method were performed at the beginning of the study and after treatment [66].

It was found that pinitol is well-tolerated and does not cause the aforementioned side effects. Treatment with pinitol significantly reduced levels of plasma glucose, insulin, fructose amine, and glycated haemoglobin (HbA1c) as well as systolic and diastolic blood pressure. Pinitol also reduced low-density lipoprotein (LDL) levels and increased the level of HDL cholesterol. Additionally, pinitol significantly reduced the LDL/HDL ratio and indicated a tendency to reduce TG and TG/HDL-cholesterol. Treatment with pinitol did not significantly affect BMI, waist circumference, body fat content, glutamic oxaloacetic transaminase, glutamate pyruvate transaminase, blood urea nitrogenorcreatinine. The authors of the study did not find literature data and did not show the correlations between different levels of these parameters and the effect of pinitol [64].

In 2007, 20 patients with T2DM (6 men and 14 women) were treated with pinitol containing 40%–60% of *chiro-*inositol from soya for 12 weeks. Additionally, patients were also receiving their drugs. Postprandial glucose and glycated hemoglobin (HbA1c) levels were significantly reduced after 12 weeks of pinitol administration. The levels of adiponectin, leptin, free fatty acids (FFA), and C-reactive protein (CRP) in plasma differed significantly before and after the administration of pinitol. Defective metabolism of DCI may be one of the causes of insulin dysfunction and its resistance in T2DM. The current study showed that 12 weeks of pinitol administration significantly reduced glucose, postprandial glucose and HbA1c levels in plasma 53].

### 7.2. Myo-Inositol and D-Chiro-Inositol

In 2016, a pilot study was conducted with patients with T2DM. This study included another group of patients with T2DM treated with at least one glucose-lowering drug, independently of gender or disease duration. Patients were qualified for clinical trials if their glucose status was consistently suboptimal for at least three months. Other criteria included age over 18 years and unchanged glucose-lowering treatment in the last three months. For all patients, combined doses of MI, DCI, and folic acid were provided twice a day as a supplement to their glucose-lowering drugs. Additionally, patients were recommended a diet with reduced glucose and low glycemia according to standard care and maintenance of standard physical activity. The study group contained 20 patients with T2DM, of which one-fourth were men and almost half of the entire group suffered from complications related to diabetes, for example, comorbidities, such as hypotensive and lipid disorders, occurred in almost half of the cases. After three months of treatment, blood glucose and HbA1c levels dropped significantly compared to the baseline. There were no statistically significant differences in systolic and diastolic blood pressure, lipid profile, or BMI level changes. None of the patients reported any adverse effects related to inositol intake. These studies indicated that inositol may be an important part of the strategy to improve glycemic control in T2DM, and its supplementation is effective in lowering fasting blood glucose and HbA1c levels [68].

Additionally, there is evidence that MI with DCI epimerase activity and the ratio of *chiro-*inositol to MI are reduced in the tissues of rats with T2DM. This mechanism may also play a role in explaining the state of insulin resistance. This ability is closely related to the various biological activities that inositol isoforms cause and MI is more effective in increasing insulin sensitivity by typical insulin-dependent tissues and DCI is more involved in tissues where glycogen synthesis takes place. Although the study was conducted among patients with T2DM, the reduction of HbA1c in this experiment was quite surprising due to the short treatment period. It was found that there is a significant effect of inositol in glycemic control. The reason for this may be the reduction of insulin resistance status due to the inositol metabolic action. However, it cannot be excluded that some of these effects may be associated only with lifestyle changes, such as more appropriate diet and physical activity implementation. This experiment was a pilot study and, despite the small study group, statistical significance in two main parameters has been achieved (i.e., in blood glucose and HbA1c) defining glycemic control [68].

Another study on pregnant mouse model was conducted in 2015 to test the effect of the MI and DCI mixture on the metabolic profile of females with metabolic syndrome and obesity during pregnancy. The mice of the study suffered from moderate hypertension and were placed on a high-fat diet for four weeks to induce phenotypes similar to the metabolic syndrome. Similarly, wild-type C57BL/6 mice were on a high-fat diet for four weeks to induce an obesity model. Then mice were bred with wild-type males. On the first day of pregnancy, the mothers were administered a mixture of MI and DCI in water or water as a placebo control. Compared to mice fed with placebo, mean systolic blood pressure was lower in mice that were treated with MI and DCI, while there were no differences in systolic blood pressure between these groups. Mice treated with MI and DCI showed lower glucose values, but no differences were found in obese pregnant mice. Leptin concentration was lower in mice treated by MI and DCI. No other differences were observed in any of the other metabolic biomarkers in tested serum. The body mass did not differ significantly but was lower in obese mice after DCI treatment in comparison with the placebo group [26].

In 2008, a study was conducted involving 17 patients who were referred for medical examinations and had been diagnosed with diseases such as obesity and hyperlipidemia, but without taking drugs. All patients received MI in a dose of 5 g per day for one week and then the dose was increased to 10 g a day for one week. The patients’ diets were not changed, although some nutritional supplements were banned during the treatment period. Blood samples were collected and measurements of weight, height, and waist circumference were recorded before and after the first and second week of treatment. Treatment with MI significantly increased the diastolic pressure as well as measured plasma parameters. In addition, LDL-related parameters also significantly lowered plasma and diastolic pressure levels and significantly lowered LDL-related parameters. Glucose tended to decline after treatment. The most significant changes included a reduction in the small dense LDL (sdLDL) level. The average level of highly sensitive C-reactive protein (hsCRP) was significantly elevated after two weeks, probably due to inflammation in one case. MI had no effect on HDL-related parameters, such as HDL-C and apolipoprotein A-I (apoA-I), or TG levels.

Patients with hyperlipidemia treated with MI were divided into two groups: Patients diagnosed as either with or without the syndrome. The group with metabolic syndrome was characterized by a much higher BMI, waist size and apolipoprotein E level as well as lower parameters associated with HDL and high levels of hsCRP, TG and sdLDL. Comparison of the MI treatment effectiveness in the group with metabolic syndrome and the group without this disease with hyperlipidemia revealed that the blood glucose level decreased only in the group with metabolic syndrome. The levels of hsCRP and sdLDL were also reduced, especially in the group of patients with syndrome. Additionally, MI treatment improved the condition of patients with obesity and hyperlipidemia but had no effect on HDL or TG levels [69].

## 8. Conclusions

Many factors affect metabolic syndrome, which is one of the most dangerous initiators responsible for myocardial infarction [58]. It has been estimated that about 20–25 % of the adult population may have metabolic syndrome and their chance of dying is two-fold higher and their chance of a heart attack or stroke is three-fold higher than patients without the syndrome [42,70]. Additionally, patients with metabolic syndrome have a higher risk of developing T2DM.

T2DM is one of the greatest challenges of modern medicine. In T2DM (unlike type 1), the body’s ability to produce insulin is preserved initially and for a long time, but the body becomes resistant to its action. Hyperglycemia, or excessively high blood glucose, appears to be due to insulin resistance [69]. This means that the tissues are not sensitive to this hormone and do not accept the right amount of glucose. To deal with high blood sugar levels, the body produces more insulin at the initial stage of the disease development and hyperinsulinemia occurs. More insulin in the body results in increased body weight and blood pressure. The pancreas produces increased amounts of insulin and its cells become depleted and destroyed over time [42,71].

Compared to other studies aimed at treating these diseases, similar studies were performed in 2002 testing the effects of conjugated linoleic acid (CLA) in metabolic disorder treatment. CLA is a polyunsaturated fatty acid that has metabolic and anti-obesity activities. The trans10cis12 (t10c12) CLA isomer seems to cause these effects, including improved insulin sensitivity. The aim of the study from 2002 was to investigate whether t10c12 CLA or a mixture of CLAs could improve insulin sensitivity, lipid metabolism or body composition in obese patients with metabolic syndrome symptoms. After treatment with CLA, insulin sensitivity decreased significantly in the group treated with t10c12CLA in contrast to insulin deficiency in CLA after treatment with t10c12CLA. A significant reduction of insulin sensitivity after treatment with t10c12CLA did not depend on age or changes in glucose level, fat, BMI, or abdominal fat levels. It has also been demonstrated that the t10c12CLA isomer significantly increases insulin resistance, fasting glucose resistance, and dyslipidemia in obese diabetic men [72].

MI and other cyclitols indicate a number of health-promoting and therapeutic properties [12,50] which may be responsible for improving lipid profiles by reducing serum triglyceride and total cholesterol after oral administration of pinitol together with MI. DP is well-researched and documented for insulin-like effects In addition, it was found that MI has antioxidant, anti-inflammatory, and anti-cancer properties [21,73,74]. All conducted studies have indicated that compounds belonging to cyclitol compounds may have a positive effect on patients with metabolic disorders: obesity, diabetes, and cardiovascular system diseases and may also reduce the risk of metabolic disorders.

## Figures and Tables

**Table 1:** Chosen cyclitols doses and their effects.

No.	Authors	Cyclitol	Object/Dose	Major In Vivo & In Vitro Effects
**1.**	Gao et al. (2016) [55]	DCI	Rats/group I: 30 mg/kg; group II: 60 mg/kg	- reduction of blood glucose level; - significant reduction of insulin level in serum - regulation of glycogen synthase and glucose transporter type-4; - increasing glucose transporter type-4 expression in skeletal muscles; - regulation of insulin-mediated glucose uptake;
**2.**	Tosti et al. (2016) [56]	DCI	Women/ 100 mg/for each test subject	- BMI reduction; - reduction of blood sugar and lipid values; - reduction of total cholesterol; - reduction of triglyceride values;
**3.**	Rengarajan et al. (2014) [34]	DP	MCF-7 cell line/20, 40, 60, 80, 100, 120 µM	- significant inhibition of MCF-7 cell proliferation in a concentration-dependent manner; - an increase in p53 and Bax and a decrease in Bcl-2 and NF-κBexpression;
**4.**	Bates at al. (2000) [57]	DP	Obese-diabetic*ob/ob* mice/100 mg/kg	- a decrease in plasma glucose level;
**5.**	Unfer et al. (2011) [7]	MI; DCI	Women/ 2 g/for each test subject	- reduction of immature oocytes in the *myo*-inositol treatment group compared to the D-*chiro*-inositol treatment group
**6.**	Moreira et al. (2018) [59]	DP	C57BL/6 mice/ 10 mg/kg	- an increase in the concentration of nitrite in blood, which was inhibited by L-NAME and calmidazolium; - reduction of systolic blood pressure;
**7.**	Plow et al. (2017) [60]	MI	LepR^db/+^ (db/+) mice/10 g/kg	- reduction of weight and fat storage; - reduction of inflammatory marker expression in adipose tissue; - a reduction in the hyperleptinemia observed in db/+ mice; - increasing insulin sensitivity and glucose uptake;
**8.**	Nordio et al. (2012) [19]	MI; DCI	Women/550 mg of MI plus 13,8 mg of DCI twice a day	- improvement of the metabolic parameters;

There are a few cases in which the initial dose of a particular drug is not available in a given species. Therefore, the choice of the initial dose of such drugs for research, experimentation, or clinical trials is a problem. It should be emphasized that widespread dose scaling based solely on body weight (mg/kg) is not an appropriate approach, because the biochemical and functional systems, particularly in pharmacokinetics, differ in different species [61,62].

## Abbreviations

BMI	Body mass index
DCI	D-*chiro*-inositol
DP	D-pinitol
FFA	Free fatty acids
GDM	Gestational diabetes mellitus
GLUT	Glucose transporter
HDL	High-density lipoprotein
IPG	Inositol phosphoglycans
LDL	Low-density lipoprotein
MI	M*yo*-inositol
PCOS	Polycystic ovary syndrome
STZ	Streptozotocin-induced diabetes
T2DM	Type 2 diabetes mellitus
TG	Triglycerides
MI-IPG	Myo-inositol phosphor glycan
